# Dietary practices and inflammatory bowel disease

**DOI:** 10.1007/s12664-018-0890-5

**Published:** 2018-09-12

**Authors:** Jimmy K. Limdi

**Affiliations:** 1 Division of Gastroenterology, The Pennine Acute Hospitals NHS Trust, Manchester, M8 6RB UK; 2 Manchester Academic Health Sciences, University of Manchester, Manchester, UK

**Keywords:** Crohn’s disease, Diet, Dietary practices, Inflammatory bowel disease, Ulcerative colitis

## Abstract

The etiology of inflammatory bowel disease (IBD) remains elusive but it is believed to result from incompletely understood interactions between environmental triggers in a potentially genetically susceptible host and a subsequent aberrant immune response. Its incidence is increasing worldwide at an unprecedented rate, outpacing what genetic influences alone could instigate. The increasingly integral role played by eating in social life has led patients to gravitate to diet and food in their consultations with physicians and other health care professionals, in an attempt to improve, control, or even “cure” IBD through diet. Diet is a modifiable factor, and both patients and healthcare professionals have fuelled resurgent interest in the role of diet in maintaining IBD remission. Despite significant and increasing interest, there is a lack of credible evidence to support dietary modification or restrictions to prevent relapse of IBD. However, recent studies have shown that more than half of the patients believe that diet plays an important role in triggering relapse, leading to self-imposed dietary restrictions, some of which can have adverse consequences. This underpins the need for physicians and health care professionals to have a better understanding of dietary practices, in triggering, perpetuating, and improving IBD. This review examines and discusses the evidence behind this.

## Introduction

The inflammatory bowel diseases (IBDs), comprising Crohn’s disease (CD) and ulcerative colitis (UC), are relapsing-remitting immune disorders of the gastrointestinal tract, hypothesized to occur from the combined effects of environmental factors in a genetically susceptible host [1, 2]. An unprecedented incidence in “developing” countries, immigrant populations into areas of high incidence and indeed globally, underpins the role of environmental factors in the etiopathogenesis of IBD [3]. The parallelism between “westernization” and particularly high dietary intakes of total fat (particularly animal fats, ω-6 polyunsaturated fatty acids (PUFA), milk fats), refined sugars, meat and lower intakes of fruit and vegetables, implicates diet in the great risk of developing IBD [4, 5]. Diet may influence intestinal inflammation through several biologically plausible mechanisms including dietary antigen presentation, alterations in the gut microbiome, the mucosal immune system and epithelial barrier function among others [2]. Nutrition, on the one hand, is essential to life, but eating has also increasingly become an integral aspect of socializing and pleasure, leading patients to gravitate to diet and food in their consultations with physicians, seeking to improve, control, or even “cure” IBD through diet [6–9]. Dietary research is fraught with challenges, not in the least including the manifold variables in dietary intake, the proportion of food intake relative to other dietary components, the potential for complex interactions between food groups, variable food metabolism among individuals, and inherent differences in food products [10]. Strong patient interest in diet in IBD, the lack of credible scientific evidence to support dietary recommendations, perceived physician indifference, or indeed variable knowledge among healthcare professionals has led patients to seek information from the lay press, internet, and other sources to address their unmet needs, potentially misleading patients through inaccurate information and fuelling self-imposed restrictions with consequent adverse effects [11, 12]. This underpins the need for physicians and healthcare professionals to have a better understanding of dietary practices, in triggering, perpetuating, and improving IBD.

## Epidemiology

IBD prevalence is highest in northern America, northern Europe and Australia increasing globally, particularly in Asia with industrialization and westernization of lifestyles and diet [1, 13, 14]. Furthermore, immigration into areas of high incidence (e.g. South Asians migrating to northern America and the United Kingdom) also increases risk of IBD [14, 15]. Intriguingly, second-generation immigrants to Sweden had a similar rate of developing IBD as native Swedish populations, emphasizing the role of early life exposures and diet in the etiology of IBD [16]. Several etiological theories link immigration and industrialization to IBD incidence and prevalence. The “hygiene hypothesis” suggests that reduced exposure to a variety of enteric organisms in early childhood from improved sanitization results in an ineffective and aberrant immune response [17]. Another theory, the “cold chain hypothesis,” argues that prolonged refrigeration of foods affects its bacterial content, promoting growth of psychotropic bacteria such as Listeria and Yersinia, both of which have been identified in patients with CD [18]. Alterations in the intestinal microbiota increase susceptibility to aberrant immune responses triggering IBD. A growing body of evidence suggests that dietary factors impact on microbiome composition and epithelial barrier function instigating and perpetuating IBD.

## Diet and the risk of IBD

The influence of diet as a risk factor for developing IBD has been studied in a number of epidemiological, observational, prospective, and retrospective case-control studies [19–24]. A common signal from large observational studies is the association of meat and fat (animal fats, ω-6 PUFA) with low and increased risk with diets higher in fiber, fruit, and vegetables [4, 24–26].

### Fat intake

A causal association between increased fat intake and IBD incidence was first suggested through the temporal association between granulomatous ileitis and the introduction of margarine early in the twentieth century [27]. A Japanese study investigated dietary changes and CD incidence and noted a strong association with animal protein intake followed by ω-6 PUFA and milk protein [19]. Margarine consumption (ω-6 PUFA) was noted to be a risk factor for UC in another Japanese study [28]. Maconi et al. noted a similar risk with margarine consumption for UC but not for CD [20]. They also noted a higher risk of CD (but not UC) with red and processed meat [20].

In the EPIC study, a higher consumption of linoleic acid (ω-6 PUFA in red meat, margarine, and cooking oils) was associated with a higher risk of developing UC whereas higher consumption of ω-3 PUFA was associated with a lower risk of UC [24]. Eating fish appears protective against IBD [5, 20, 23]. A study reporting on children newly diagnosed with CD found a lower risk with reported fish consumption a year before diagnosis [5]. A marginally positive association was noted between total PUFA consumption, including ω-3 and ω-6 PUFA at baseline and the risk of developing IBD [23]. A higher ratio of ω-3 to ω-6 fatty acids was also noted to confer a lower risk of UC in the Nurses’ Health Study (NHS) [25]. It appears that early life exposures (20 years or more before onset) are associated with the risk of developing IBD and CD in particular [25, 26]. A systematic review of 19 case-control studies assessing pre-illness diets and IBD risk linked high-fat consumption to the risk of developing IBD [4].

### Omega-3 fatty acids

Dietary fat comprises saturated and unsaturated fats, the latter consisting of polyunsaturated fatty acids (PUFA) and monounsaturated fatty acids (MUFA) [29]. The essential fatty acids (ω-3 and ω-6 PUFA) cannot be produced in the human system and must be supplied through the diet. They are inflammatory regulators in the prostaglandin and eicosapentaenoic acid pathways playing a role in cell signaling, with ω-3 PUFA (from oily fish, e.g. salmon and mackerel, and α-linolenic acid from plants) being anti-inflammatory and ω-6 PUFA (vegetable oils, e.g. safflower, corn, and cottonseed oil) being pro-inflammatory. An equipoise is needed between these for homeostasis [29]. Western diets are typically high in ω-6 PUFA and lower in ω-3 PUFA conferring a greater risk of inflammatory disease including IBD [29]. Attempts to augment ω-3 intake have demonstrated a reduction in inflammatory parameters but no effect on disease activity or relapse rates [30, 31]. Two placebo-controlled trials did not show the benefit of ω-3 supplementation for maintaining remission in CD but showed significant benefit for UC [32]. An interesting observation in a systematic review was that a ratio of ω-3/ω-6 of 1 was associated with a significantly higher remission rate [33]. Another systematic review assessing ω-3 supplementation for the treatment of active UC showed inconclusive results, limited by marked heterogeneity between studies [34]. Variable bioactivity of the ω-3 supplements used may have affected response.

### Protein intake

The risk of IBD with meat consumption has been reported with remarkable consistency across other cohorts. A large French prospective cohort study of 67,581 women aged 40–65 years found that animal protein intake was associated with UC risk [23]. In the European Prospective Investigation into Cancer and Nutrition (EPIC) study, the highest quartile of linoleic acid (ω-6 PUFA) present in red meat, margarine, and cooking oils (sunflower and corn oil) was associated with an increased risk of UC [28, 35]. Red and processed meat, protein, and alcohol were associated with a risk of UC relapse in another prospective cohort study, which also suggested that meat consumption may increase the risk of relapse [36]. A high risk for CD and UC was observed with higher processed meat intake in monozygous and dizygous twins [37]. Although mechanistic explanations for protein-induced changes promoting IBD need to be elucidated further, a working hypothesis is that protein metabolites provide substrates for gut bacteria affecting microbiome composition or short-chain fatty acids (SCFA) affecting enterocyte function [36]. Another postulated mechanism by which red meat can exacerbate IBD is the sulfur and high cysteine content, used by sulfate-reducing bacteria to generate hydrogen sulfide (H_2_S), which in turn has detrimental inflammatory effects [38]. This area needs further study.

### Carbohydrates

Carbohydrates are classified by their degree of polymerization into monosaccharides and disaccharides (sugars-glucose, fructose, and sucrose), oligosaccharides (galactooligosaccharides, fructooligosaccharides), and polysaccharides such as starch, cellulose, and inulin. Simple sugars and starch (“available” carbohydrates) are hydrolyzed and absorbed in the small intestine. “Unavailable” or resistant starches, such as inulin, galactooligosaccharides, and fructooligosaccharides, cannot be hydrolyzed in the small intestine and are fermented by the microbiota in the large intestine [39]. Insoluble fibers such as bran and cellulose, on the other hand, transit through the intestinal tract intact, adding bulk to intestinal contents and providing a laxative effect [39]. It is becoming increasingly clear that it is the type of carbohydrate consumed that has a beneficial or detrimental effect on IBD. A higher consumption of “available” carbohydrates such as glucose, fructose, sucrose, or lactose may exceed intestinal absorptive ability and increase luminal sugar content as also osmotic load. Gut microbiota may use this as an energy substrate. Indeed, there is an increased prevalence of fructose and lactose malabsorption in patients with IBD [40]. High consumption of sugar has been shown to increase dysbiosis and gut permeability with consequent inflammation in mouse models, a scientific basis for the popularized fermentable oligo-, di-, and monosaccharides and polyol (FODMAP)-restricted diet, used variably in IBD patients and which excludes fermentable oligo-, di-, and monosaccharides and polyols [41, 42]. A high pre-illness (IBD) intake of refined sugars and low fiber has been observed in numerous studies [4, 21, 43] but two large observational cohorts found no association between baseline sugar intake and IBD [22, 44]. Fruit consumption appears to be associated with a decreased risk of developing CD but not for UC [4, 5, 21, 45, 46]. This association was stronger in those who consumed fruit more than four times a day than those consuming fruit less than once a day [4].

Dietary fiber may have a protective role [4, 21, 27]. Non-digestible carbohydrates (prebiotics) are fermented in the colon (bypassing small intestinal absorption) and have a role in promoting epithelial integrity, bacterial diversity, and production of SCFA’s, butyrate, acetate, and propionate. Butyrate is an important source of energy for colonocytes and has anti-inflammatory effects through reduction in NFκB activity and increasing apoptosis in colon cancer cells [39]. Higher fiber consumption was associated with decreased CD risk and also probably protects against colorectal cancer [4, 5, 21, 47]. Phytochemicals (lignans, flavonoids, and antioxidants) in fruit, cereals, and vegetables exert anti-inflammatory effects through growth factors, maintenance of intestinal barrier integrity, and through an antioxidant effect [39, 48, 49]. Indeed, the consumption of fruit and vegetables has been associated with reduction in biochemical inflammatory markers (CRP, TNF, and IL-6) [50]. The EPIC study found no protective effect of dietary fiber against the risk of developing UC [23]. Retrospective studies have observed a protective effect from fruit and vegetables against IBD [39]. Recent data suggest that a higher intake of fiber may reduce the risk of flares in patients with CD but not UC [51]. It is agreed that dietary fiber need not be restricted in IBD patients in remission and in the absence of strictures [52, 53].

### Vitamins D, C, and E

There has been resurgence of interest in recent years in the pro-hormone vitamin D beyond its classical role in bone metabolism, recognizing its effects and additional benefits in immune regulation, promoting innate immunity through synthesis of antimicrobial proteins cathelicidin and certain defensins [54]. The higher incidence of CD in northern countries with lower exposure to sunlight postulates a role for hypovitaminosis D in the etiology of IBD [55]. The active metabolite, 1,25-dihydroxy vitamin D, binds to the vitamin D receptor in tissues including immune cells, thereby modulating gene expression [54]. Vitamin D deficiency worsens colitis through a myriad of mechanisms and alters the gut microbiome [56]. In the NHS, higher predicted plasma levels of 25-hydroxyvitamin D (25[OH]D) were associated with a significantly reduced risk for incident CD and non-significantly reduced risk for UC in women [57]. A randomized, placebo-controlled trial tested the efficacy of dietary supplementation of 1200 IU vitamin D3 administered daily for 12 months to patients with CD in remission [58]. Supplementation reduced the risk of clinical relapse from 29% to 13% [58]. Patients should be calcium-replete before supplementation as 1,25 OH D can mobilize calcium from bone [54].

Supplementation with vitamins C and E has not been shown to be of clinical benefit although they do appear to have effects on biomarkers of oxidative stress [59].

### Curcumin and other antioxidants

Curcumin is a natural phenol, derived from turmeric, a spice commonly used in Indian cooking and has been used in many inflammatory and immune-mediated diseases [60]. It has been shown to inhibit nuclear factor-κB, signal transducer and activator of transcription 3 (STAT-3), p38 mitogen-activated protein kinase, and T helper-1 cytokines [60]. Curcumin has been shown to be pro-apoptotic and has been studied in a variety of chronic immune-mediated diseases [61]. Two randomized, placebo-controlled trials have demonstrated evidence of benefit with curcumin in mild-moderately active UC [62, 63]. The addition of 3 g/day of curcumin was superior to placebo for the induction of clinical remission and maintenance of clinical response and reduction in mucosal inflammation at endoscopy [63]. A dose of 1 g/day of curcumin in addition to sulfasalazine or mesalazine was superior to placebo in maintaining remission in UC [62]. Notably, pure curcumin preparations were used in the trials, which cautions against extrapolation of these results, albeit encouraging and emphasizing the need for further studies before clear recommendations can be made [61].

Polyphenols such as blueberries and tea extracts have anti-oxidant and anti-inflammatory effects and appear attractive options to prevent or treat chronic inflammatory diseases. Although animal models of colitis provide evidence that polyphenols can effectively modulate intestinal inflammation by modulating cell-signaling pathways, they need confirmation in human IBD [64].

## Dietary perceptions and practices in patients with IBD

It is evident from the above discussion that despite significant strides in our understanding of the role of diet within the complex “exposome” of IBD, the variability in dietary intake and recall, the complex interactions between food groups and variable food metabolism among individuals lead to heterogeneity in human studies and continue to drive the complexities of nutritional research. The limitations posed by conflicting scientific literature and gaps in knowledge have unwittingly fed into the several (and often harmful) myths and misconceptions held by patients, seeking to nourish their information needs from “unqualified” sources [12, 65]. Despite strong patient interest in diet as trigger or treatment in IBD, few studies have evaluated patient’s dietary beliefs and practices [6–8, 66, 67].

Hou et al. observed that patients frequently derived dietary advice from Internet-based sources, and these involved placing food restrictions [8]. A cross-sectional study of 1647 IBD patients from the Crohn’s and Colitis Foundation of America (CCFA) partners cohort noted a high prevalence of gluten avoidance in the absence of celiac disease, two thirds describing symptom improvement and a third reporting fewer IBD flares [67]. A variable presence of celiac disease has been described in IBD, and testing for celiac disease may be appropriate in IBD patients, particularly in those with iron deficiency and medically refractory disease, in whom identification and gluten avoidance would be beneficial [68]. Increasing evidence and understanding of the role of gluten in activating innate immune responses and abrogating the intestinal barrier suggests that gluten avoidance may, in fact, be reasonable for symptomatic patients although proof of principle is needed before this approach can be recommended with robust scientific and clinical evidence [68].

In a study of UK, nearly 50% IBD patients perceived diet to be an initiating factor in their disease and 57% believed that dietary factors could trigger a relapse [6], similar to observations by Zallot and colleagues [7] in a French study. Patients often modify their diet, typically avoiding fatty and spicy foods, fruit, vegetables, alcohol, and milk often during remission as also “to prevent” relapse [6–8, 69, 70]. It is noteworthy that cruciferous vegetables, fruit peels, nuts, and seeds have an insoluble fiber that is metabolically fermented in the colon and increases colonic transit. The recently popularized FODMAP-restricted diet may provide a mechanistic explanation for the symptomatic benefit, noted through a reduction in osmotic effect and fermentation by intestinal bacteria, in avoiding such foods and are discussed later in this review [41, 42]. Dietary restrictions while beneficial in some circumstances may prove detrimental in others. Lactose intolerance may be present in IBD patients, but it may still be possible to consume dairy produce with lower lactose content, such as cottage cheese and butter as also yoghurt, because of live cultures that produce their own lactase. “Complete” avoidance of dairy products has the potential for calcium and vitamin D deficiency [6, 68]. Significantly lower intake of vitamins C, E, and K and dairy products were noted in another study [71]. The potential for irrational dietary exclusions to impact negatively on health cannot be underestimated and underpin the need for patient education on diet, limitations with knowledge notwithstanding. Dietary counseling is shown to be effective in managing and preventing nutritional deficiencies in patients with IBD [59].

Despite this, access to dietary information is lacking even from recent studies [6, 7, 66]. In stark contrast to where patients actually access information (internet based sources), they seek information from health care professionals, gastroenterologists, dieticians, and nurse specialists [6, 12, 66, 70].

Patient education and disease-related knowledge are a key determinant of the clinician-patient relationship and a key outcome modifier in chronic disease [12].

## Defined and exclusionary diets

Patients with IBD have a strong interest in dietary modification as part of their holistic approach to managing chronic disease and often turn to popular “exclusionary” diets for control or “cure” of “disease” [4, 6–8]. The potential for macronutrient restriction to result in nutritional deficiencies or indeed malnutrition underpins the need for gastroenterologists, dieticians, and nurse specialists to have a critical appreciation of the literature enabling a considered approach differentiating anecdotal success from credible science [8, 68, 70]. Described below are some of the popular defined and exclusionary diets, emphasizing (but not advocating) the philosophy, science, and nutritional impact of these interventions on IBD.

### The specific carbohydrate diet

The specific carbohydrate diet permits the consumption of monosaccharides (glucose, fructose, and galactose) but excludes complex carbohydrates [8]. Thus, honey, fresh fruits, and vegetables (with the exception of potatoes and yam) and homemade yoghurt are permitted. Legumes such as lentils and split peas are also permitted but chickpeas and soybeans are not. Grains, canned fruits, and vegetables are not allowed as also milk due to its lactose content although lactose-free cheeses are allowed [8]. Processed, smoked, and canned meats are also not permitted owing to possible contamination by sugars and starch additives [8]. The rationale behind exclusion of complex carbohydrates is that they are poorly absorbed in the digestive tract and consequently cause bacterial fermentation (Table 1). Abrogation of the intestinal epithelial barrier then promotes and perpetuates intestinal inflammation and injury [8, 41, 68]. The specific carbohydrate diet (SCD) has shown promise in pediatric studies with evidence of improvement in clinical symptoms, clinical scores, and mucosal improvement within 12 weeks of initiation and also demonstrating evidence of an increase in bacterial diversity, justifying further studies to better understand and define its role [72]. A recent systematic review examining dietary interventions including restrictions noted promising results with SCD [73].

**Table 1:** The specific carbohydrate diet

Principle: Disaccharides and polysaccharides are poorly absorbed in the human digestive tract, results in bacterial and yeast overgrowth with the overproduction of mucous. It only permits simple carbohydrates		
Food group	Include	Exclude
Fruit	All fresh fruit	Canned fruit
Vegetables	Fresh vegetables	Canned or frozen vegetables Potato, yam, legumes, corn
Grains	None	Avoid all cereal grain
Protein	Fresh meat, lentil, split pea	Canned, processed or smoked meat Legumes (chickpea, soybean)
Dairy	Only lactose-free	All dairy products, soybean milk Instant tea and coffee
Beverage	Wine	Beer
Other	Honey, butter	Corn syrup, margarine, chocolate

### The lactose-free diet

Lactose malabsorption is the impaired ability to digest lactose as a result of a reduction in production of the enzyme lactase. Lactose malabsorption appears to be more common in CD patients, with the highest prevalence in Asians and Native Americans; moderate prevalence in Afro-Caribbean, Southern European, Hispanic, and Jewish populations; and lowest in Northern and Western Europeans [74]. The observation by our group and others that IBD patients avoid dairy produce is of relevance to clinicians [6, 68]. Although lactose intolerance may be present in IBD patients, it may still be possible for patients to consume dairy produce with lower lactose content, such as cottage cheese and butter as also yoghurt, because of live cultures that produce their own lactase [68]. Deficiency of calcium and vitamin D from IBD disease activity, its treatment with corticosteroids, and “complete” avoidance of dairy produce should be addressed by clinicians and dieticians. Although avoidance of lactose may improve symptoms in those with lactose intolerance, evidence for a mechanistic effect and objective disease improvement is currently lacking.

### The anti-inflammatory diet

The IBD-anti-inflammatory diet (AID) is based somewhat loosely on the SCD on the premise that certain carbohydrates act as substrates to pathogenic bacteria inducing dysbiosis in the gut lumen and thereby limits the intake of refined sugars, gluten-based grains, and complex carbohydrates with pro-inflammatory effects on the gut [75]. It encourages the intake of prebiotics and probiotics (e.g. leek, legume, and fermented food) to restore intestinal flora, advocating against saturated fat intake and promotes intake of ω-3 rich foods [75]. In a case series, 100% of the patients reported symptom improvement in 4 weeks [75]. The hypothesized mechanisms deserve further study.

### The low FODMAP diet

The potential for higher consumption of “available” carbohydrates such as glucose, fructose, sucrose, lactose, or polyols to exceed intestinal absorptive ability and to increase luminal sugar content as also osmotic load has been recognized [41, 42]. Resultant increased intestinal permeability may act as a predisposing factor for IBD in susceptible individuals [41, 42]. Common symptoms associated with a high FODMAP intake include abdominal bloating and distension, flatulence, crampy pain, and diarrhea [41] and are arguably more pronounced in patients with irritable bowel syndrome (IBS) than IBD but influence symptoms in both. A low FODMAP diet involves elimination of foods high in FODMAPs for 6–8 weeks, followed by gradual re-introduction under specialist dietician supervision and thereby determining individual susceptibility to food groups that exacerbate symptoms (Table 2) [41]. A retrospective study demonstrated positive effects of a low FODMAP diet in IBD patients in remission [42]. The complexity of a restrictive diet may pose challenges to adherence. Post-secondary level education and working under 35 h/week were identified as predictors for long-term adherence [42]. Further studies exploring its effects on gut microbiota, intestinal permeability, and inflammation are warranted as also studies involving effects on hard endpoints such as mucosal healing and the potential for nutritional deficiencies from dietary restriction.

**Table 2:** The low-FODMAP diet

Principle: Foods containing fermentable oligosaccharides, disaccharides, monosaccharides, and polyols are poorly absorbed, presents a high osmotic load and are rapidly fermented by bacteria in the gut which can cause Irritable bowel syndrome and Inflammatory bowel disease symptoms		
Food group	Include	Exclude
Fruit	Bananas, strawberry, raspberry, blueberry, orange, mandarin, Clementine, cantaloupe, grapes, melons(honeydew), lemon, lime, kiwi, passion fruit	Apple, applesauce, apricots, blackberries, cherries, nectarines, pears, peach, plum, prune, watermelon, grapefruit, dried fruit
Vegetables	Carrots, celery, corn, alfalfa, bean sprouts, bell pepper, broccoli (< 1/2 cup), Brussels sprout (< 2 sprouts) bok choy, cucumber, eggplant, green bean, kale, lettuce, potato, spinach, spring onion (green top), squash, tomato, turnip, zucchini	Brussel sprouts, asparagus, avocado, beetroot, cauliflower, cabbage, garlic, leek, mushroom, onion, pea shallot, snow pea, sweet corn, sweet potato
Grains	Rice, oats	Wheat, Rye
Protein	All	None
Dairy	Lactose-free yoghurt and milk, almond, coconut, rice or soy milk, hard cheese, low-lactose cheese	Cow, goat, sheep milk, buttermilk, soymilk, soft cheese cream and ice cream
Beverage	Fruit juice and vegetable juices from permitted foods, wine, vodka, gin	Soft drinks, sports drinks, white tea, green tea, coconut water
Other	Maple syrup	Honey and sweeteners

### The Paleolithic diet

The agricultural age, from an evolutionary perspective, has brought a paradigm change to human diet and lifestyle, with the introduction of refined sugars and grains. Its parallelism, however, with the emergence of chronic diseases such as diabetes, coronary heart disease, and indeed IBD is the basis for the Paleolithic diet, which hypothesizes that modern diets and agricultural methods have outpaced the ability of the human digestive tract to “process” such foods. Thus, it restricts complex carbohydrates (cereals, refined sugars, dairy products, potatoes, refined oils) and processed foods as also domesticated sources of meat on the premise that grain-based feed use to raise such livestock have role in perpetuating disease (Table 3) [4, 68]. It advocates the intake of 50% to 65% of calories to come from plant sources and 35% to 45% from animal sources (lean meat), preferring fish over meat [68].

**Table 3:** The Paleolithic diet

Principle: The human gastrointestinal tract is poorly evolved and unable to handle diets that result from modern agricultural methods. Exposure to foods not consumed at the time of evolution may thus result in modern diseases		
Food group	Include	Exclude
Fruit	All	None
Vegetables	All except (see under exclude)	Potatoes, legumes, corn, yam, beet, butternut squash
Grains	Cereal grains	All other
Protein	Lean meats: game, fish	Processed meat, domesticated meat
Dairy	None	All dairy produce
Beverage	All others	Fruit juice, all alcoholic and non-alcoholic beverages
Other	Honey	Refined sugars

While the prospect of “rewinding the clock” with dietary practices advocated by the Paleolithic diet, when these diseases (possibly) did not exist, may seem attractive, the lack of a mechanistic explanation defining its role in triggering and perpetuating gut inflammation and disease limits our ability to make evidence-based recommendations presently.

## Conclusions

The unprecedented global rise in incidence in IBD has clearly outpaced what genetic predisposition alone could instigate placing emphasis on environmental triggers in the etiology of the disease. Compelling evidence from laboratory and clinical studies implicates dietary changes as a factor within the complex “exposome” of IBD. Dietary research in IBD is fraught with challenges from ranging the complexity of inflammation biology and its myriad triggers but also by the multiple putative mechanisms by which food components affect the gut and induce symptoms. Strong patient interest will serve as an appropriate impetus and driver for more meaningful research, which has also been outpaced by popularized diets, some with and others without credible evidence leaving our patients and healthcare professionals vulnerable from unfounded literature. It is imperative for healthcare professionals advising patients with IBD to be conversant with the strengths and limitations of current literature to ensure credibility. Meanwhile, the evidence for exclusive enteral nutrition in reducing symptoms and mucosal inflammation holds promise for the therapeutic benefit for certain diets and derived supplements. These observations will serve to optimize clinical studies through identification of nocebo effects and pro-inflammatory food groups, enabling deeper mechanistic insights into the effects of food on the microbiota and the immune system. It is plausible then, that in unraveling the mysteries of the microbiome, diet, and immune interactions, we may discover who “we are” through what we eat.
